# Integrative analysis across metagenomic taxonomic classifiers: A case study of the gut microbiome in aging and longevity in the Integrative Longevity Omics Study

**DOI:** 10.1371/journal.pcbi.1013883

**Published:** 2026-01-12

**Authors:** Tanya T. Karagiannis, Ye Chen, Sarah Bald, Albert Tai, Eric R. Reed, Sofiya Milman, Stacy L. Andersen, Thomas T. Perls, Daniel Segrè, Paola Sebastiani, Meghan I. Short

**Affiliations:** 1 Institute for Clinical Research and Health Policy Studies, Tufts Medical Center, Boston, Massachusetts, United States of America; 2 Department of Medicine, Tufts University School of Medicine, Boston, Massachusetts, United States of America; 3 Clinical Translational and Science Institute, Tufts Medical Center, Boston, Massachusetts, United States of America; 4 Bioinformatics Program, Faculty of Computing and Data Science, Boston University, Boston, Massachusetts, United States of America; 5 Department of Immunology, Tufts University School of Medicine, Boston, Massachusetts, United States of America; 6 Data Intensive Studies Center, Tufts University, Medford, Massachusetts, United States of America; 7 Department of Medicine, Albert Einstein College of Medicine, New York, New York, United States of America; 8 Department of Medicine, Geriatrics Section, Boston University Chobanian & Avedisian School of Medicine, Boston, Massachusetts, United States of America; 9 Department of Biology, Boston University, Boston, Massachusetts, United States of America; Western University, CANADA

## Abstract

There are various well-validated taxonomic classifiers for profiling shotgun metagenomics data, with two popular methods, MetaPhlAn (marker-gene-based) and Kraken (k-mer-based), at the forefront of many studies. Despite differences between classification approaches and calls for the development of consensus methods, most analyses of shotgun metagenomics data for microbiome studies use a single taxonomic classifier. In this study, we compare inferences from two broadly used classifiers, MetaPhlAn4 and Kraken2, applied to stool metagenomic samples from participants in the Integrative Longevity Omics study to measure associations of taxonomic diversity and relative abundance with age, replicating analyses in an independent cohort. We also introduce consensus and meta-analytic approaches to compare and integrate results from multiple classifiers. While many results are consistent across the two classifiers, we find classifier-specific inferences that would be lost when using one classifier alone. Both classifiers captured similar age-associated changes in diversity across cohorts, with variability in species alpha diversity driven by differences by classifier. When using a correlated meta-analysis approach (AdjMaxP) across classifiers, differential abundance analysis captures more age-associated taxa, including 17 taxa robustly age-associated across cohorts. This study emphasizes the value of employing multiple classifiers and recommends novel approaches that facilitate the integration of results from multiple methodologies.

## Author summary

The human gut contains communities of microbes that play crucial roles in health and disease. Identifying these microbes from their sequenced DNA is essential for understanding their contributions to a variety of conditions and diseases including aging. Metagenomics studies use computational tools (“classifiers”) to identify microbial species and quantify their abundances based on the DNA present in a sample. However, the tools developed use different strategies and can produce results that disagree, presenting challenges for identifying consistent findings. To address these challenges, we performed an analysis in which we used two different classifiers to investigate changes in gut microbial communities with age in two studies of extreme human longevity. Our study suggests ways to compare and mathematically combine results from multiple classifiers. One such method is a new type of meta-analysis—an analysis that combines evidence from multiple studies—which we used to account for different measurements across classifiers from the same individuals. Overall, we show that, while different classifiers generally produce results that agree, using multiple methods and combining their results enables the discovery of age-associated microbes that would not be found with a single classifier alone.

## Introduction

High-throughput sequencing approaches, such as shotgun metagenomics, have greatly increased our ability to investigate changes in microbial communities of the human gut [1]. A variety of taxonomic classification methods are available for processing shotgun metagenomics sequencing data into taxonomic profiles of microbial communities. Taxonomic profilers identify taxa and their relative abundances in each sample based on the classification of sequencing reads against a reference database. The methods underlying these classification tools vary and include marker-gene-based methods (e.g., MetaPhlAn [2], mOTUs [3]), k-mer-based approaches (e.g., Kraken [4,5], Bracken [6,7], Centrifuge [8]), and protein-based methods (e.g., Kaiju [9], DIAMOND [10]), each with different strengths.

Two popular methods, MetaPhlAn and Kraken, have been consistently used in metagenomics studies, including in the context of aging. MetaPhlAn performs classification of sequencing reads by aligning reads against a curated database of marker genes specific to taxonomic groups, with species relative abundances estimated based on clade-specific coverage [2,11]. Although this method does not use all the sequencing data, MetaPhlAn has been shown to have high specificity and high coverage of the human gut microbiome [2,12]. Kraken performs k-mer based classification by mapping individual reads to taxa via k-mer “voting” methods [4]. More specifically, Kraken maps reads to the lowest taxonomic group in the taxonomic hierarchy that shares that k-mer to infer taxonomic abundance, with estimation of relative abundances at a specific level performed by Bracken [4,6]. Although the Kraken/Bracken approach has greater sensitivity than marker-based methods and uses all the sequencing data, it has been shown to be prone to false positives [12–15]. These differences between classification approaches can greatly impact the identification of taxa, their estimated relative abundances, and downstream analyses [11,16].

Because of the differences and complementary strengths of various taxonomic classification approaches, previous work has suggested that consensus-based methods may be desirable for robust microbiome analysis [14,17,18]. However, there is little guidance or examples of this in practice. Tools, such as MetaMeta [19], WEVOTE [20], FlexTaxD [21], and a recently developed *merging strategy* using a weighted voting approach [22] have provided avenues for analysis of metagenomics data via multiple taxonomic classification methods. These integrative approaches use a variety of methods to combine classifier-specific profiles and improve accuracy of taxonomic identification. However, these methods are limited by the range of classification tools supported—e.g., MetaMeta cannot support MetaPhlAn, FlexTaxD can only support k-mer based classifiers, *merging strategy* was tested on a limited number of profilers excluding marker-gene based methods—and some methods such as MetaMeta or WEVOTE are not maintained by the developers. Additionally, these approaches all perform integration at the taxonomic classification level, creating a single combined feature table for downstream analysis. Combined classification in this way precludes comparison of taxonomic profiles and downstream findings from each profiler method. Finally, despite the availability of some tools and the potential benefits of analysis with multiple classifiers, most studies continue to rely on a single taxonomic profiler [17,23,24].

Motivated by the interest in comparing analysis results from different classifiers and leveraging their complementary strengths, we used two taxonomic profiling approaches in parallel (Kraken2 and MetaPhlAn4) to discover diversity trends and individual taxa associated with age in two studies of extreme human longevity. We performed shotgun metagenomics sequencing of the gut microbiome from individuals enrolled in the Integrative Longevity Omics Study (ILO), a new cohort study of centenarians in North America and their offspring. In addition, we acquired publicly available metagenomic sequence data from a cohort of Han Chinese individuals as a replication cohort [25]. We present this analysis as a case study to show the value of using complementary approaches to analyze metagenomics data and introduce methods, including a novel correlated meta-analysis approach, AdjMaxP, that can help integrate results across taxonomic classifiers and provide comprehensive analysis of the data.

## Results

### Two popular taxonomic classifiers detect different taxonomic profiles

**Fig 1:**
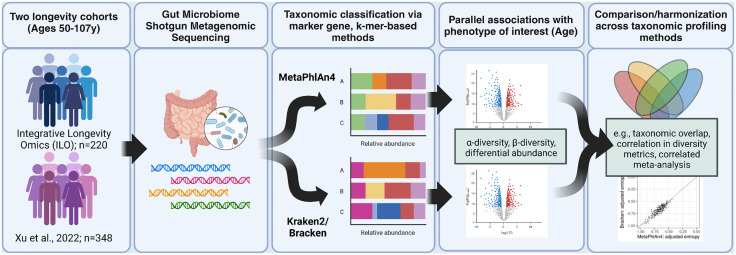
Overview of analysis pipeline for shotgun metagenomics analysis using complementary taxonomic classification approaches. Shotgun metagenomics sequencing data of the gut microbiome was generated from individuals of the Integrative Longevity Omics Study (ILO), and acquired from a cohort of Han Chinese individuals as a replication cohort. We used two complementary taxonomic profiling approaches in parallel (Kraken2 and MetaPhlAn4) to discover taxa associated with age from the two studies of extreme human longevity. We measured associations of alpha and beta diversity with age, as well as differential abundance of taxa with age, using profiles generated by both classifiers in both cohorts. We used taxonomic overlap, correlation, Procrustes analysis, voting methods, and a correlated meta-analysis approach recently developed in our research group to measure agreement and integrate downstream results across classifiers. Created in BioRender. Short, M. (2025) https://BioRender.com/d16j755.

We generated shotgun metagenomics sequencing data of the gut microbiome from 220 participants of the Integrative Longevity Omics (ILO) study (59–107 years), who were of North American/European descent and included 78 centenarians (100–107 years) and 142 of their biological offspring (59–99 years) (Fig 1 and see S1 Text). We also obtained a shotgun metagenomics sequencing dataset of the gut microbiome from 348 individuals of Han Chinese descent (50–105 years), with 116 considered to be of advanced age (90–105 years) including 13 centenarians (100–105 years), and 232 of their offspring (50–79 years) [25] (Fig 1). We processed both raw sequence data sets using KneadData [26], and performed taxonomic classification using 1) MetaPhlAn4 [2] and 2) Kraken2 ^[^4^]^ followed by Bracken [6].

There were differences in species numbers between the databases used by MetaPhlAn4 (20789 species) and Kraken2/Bracken (23127 species), with relatively minimal overlap (6292 species in common, or 27–30% of species in each database) (Table 1). After taxonomic classification and filtering procedures were applied to the cohort datasets, Kraken2/Bracken identified more species (ILO:1044, Xu:1504) than MetaPhlAn4 (ILO:787, Xu:898). The within-cohort overlap in the species identified by the two profilers was low (ILO:335 (22.4%), Xu:442 (21.3%)), and greater overlap observed within the same profiler across cohort (MetaPhlAn4:626 (59.1%), Kraken2/Bracken:846 (49.7%)). We observed similar trends across most taxonomic levels (S1 Table).

**Table 1:** Number of species identified by MetaPhlAn4 and Kraken2/Bracken.

Classification	Reference DB^a^	ILO cohort^b^	Xu et al. cohort^c^	Cohort Overlap^d^
**Kraken2/Bracken**	23127	1044	1504	846
**MetaPhlAn4**	20789	787	898	626
**Method Overlap** ^**e**^	6292	335	422	281

Table 1 displays number of species available in Kraken2 and MetaPhlAn4 reference databases and number of species identified in ILO and Xu et al. cohorts after taxonomic classification when using Kraken2/Bracken and MetaPhlAn4.

^a^ Number of species available in each classifier’s database.

^b^ Number of species identified in ILO cohort.

^c^ Number of species identified in Xu et al. cohort.

^d^ Number of species that are in-common between cohorts when using each classifier.

^e^ Number of species that are in-common between the two classifiers.

Since we found differences in the species identified across classifiers, we next investigated if the two methods differed by their quantification of relative abundances among species identified by both classifiers. The taxonomic profiles generated by Bracken and MetaPhlAn4 varied substantially based on the composition of in-common species identified by both classifiers. These shared species comprised the bulk of the sample profiles when using Bracken, representing an average of 88.1% of the total relative abundance in sample profiles in the ILO cohort, and 90.0% in the Xu et al. Cohort. When using MetaPhlAn4, shred species comprised a smaller proportion of the total, making up, on average, 68.7% of the total relative abundances in the sample profiles in the ILO cohort, and 68.3% in the Xu et al. cohort (S2A, S2B Fig). In other words, we found that MetaPhlAn4 identifies a smaller number of species that Kraken2/Bracken does not identify, but that these species are more abundant. Kraken2/Bracken identifies more unique species, which are of lower abundance on average. It is worth noting that the most abundant species in each profile were similar across classifier methods (S2C, S2D Fig), suggesting that the dominant taxa are similar regardless of the classifier used, and differences may result primarily from differential identification of lower abundance taxa. In addition, we investigated the unique taxa in each cohort dataset that did not overlap between taxonomic classifiers (Table 2 and S1 Table). Of the species found only by Kraken2/Bracken (ILO:709 species; Xu:1082 species), 61.2% (ILO) and 52.7% (Xu) were present in the MetaPhlAn4 database -- i.e., MetaPhlAn4 had the ability to identify those species but did not. In contrast, of the species found only by MetaPhlAn4 in each cohort dataset (ILO:452 species; Xu:476 species), only 1.6% (ILO) and 2.3% (Xu) were also present in the Kraken2/Bracken database -- i.e., Kraken2/Bracken had the ability to identify those species but did not. This suggests that Kraken2/Bracken has higher sensitivity than MetaPhlAn4 and/or potentially identifies more false positives, as has been suggested previously [12,15]. We observed similar trends across most taxonomic levels (S1 Table).

**Table 2:** Number of species identified by one classifier and their availability in the other classifier’s database.

	ILO cohort			Xu et al. cohort		
Classification	Unique taxa^a^	In Other DB^b^	Not in Other DB^c^	Unique taxa^a^	In Other DB^b^	Not in Other DB^c^
**Kraken2/Bracken**	709	434 (61.21%)	275 (38.79%)	1082	570 (52.68%)	512 (47.32%)
**MetaPhlAn4**	452	7 (1.55%)	445 (98.45%)	476	11 (2.31%)	465 (97.69%)

Table 2 displays number of species uniquely identified by one classifier method and the number of those species that are present or not present in the other classifier’s database.

^a^ Number of species uniquely identified by one classifier method.

^b^ Number of unique species identified by one classifier method that are also present in the other classifier’s database.

^c^ Number of unique species identified by one classifier method that are not present in the other classifier’s database.

### Age associations with alpha diversity vary based on taxonomic level and classifier

**Fig 2:**
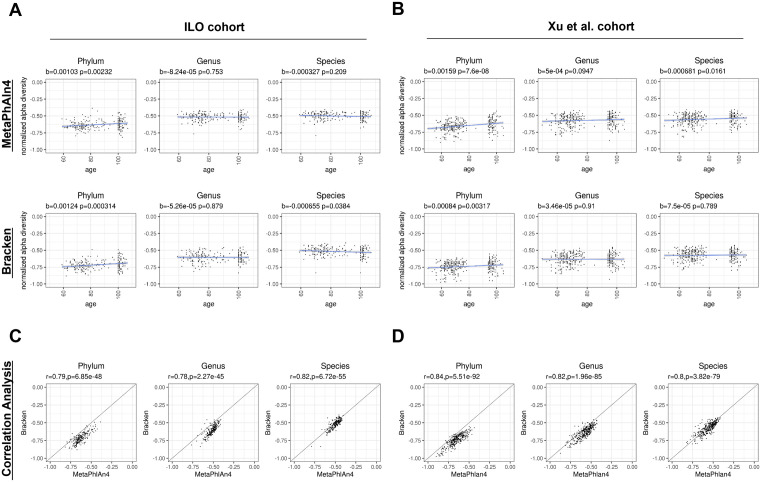
Alpha diversity displays similar changes with age at higher taxonomic levels and varies at lower taxonomic levels across classification approaches. **(A-B)** Scatterplots of the normalized alpha diversity score for each sample with age, comparing across phylum, genus, and species taxonomic levels within cohorts and between cohorts and classification methods. We employed linear regression models to evaluate the association with age, with a significance threshold set at 0.05. **(C-D)** Scatterplots comparing the normalized alpha diversity scores of samples based on classification method within each cohort. We employed the Pearson correlation analysis to evaluate differences in the sample normalized alpha diversity scores between methods, with significance threshold set at 0.05.

We calculated a normalized alpha diversity of the taxonomic relative abundances at each taxonomic level using both classifiers (S2 Table and S1 Text). In both ILO and Xu et al. cohorts, we observed a significant increase of normalized alpha diversity with age at the phylum level with both classifiers (ILO MetaPhlAn4: slope = 0.00103, p = 0.00232; ILO Bracken: slope = 0.00124, p = 0.000314; Xu MetaPhlAn4: slope = 0.00159, p = 7.6e-8; Xu Bracken: slope = 0.00084, p = 0.00317) (Fig 2A, 2B). This trend in diversity with age persisted at the class and order levels of the taxonomy (S3A, S3B Fig). At the genus and species level, the increasing trend of normalized alpha diversity with age was not observed (Fig 2A, 2B). The results varied across classifier and cohort, with two cases at the species level showing significance although having flatter trends (ILO Bracken: slope = 0.000655, p = 0.0384; Xu MetaPhlAn4: slope = 0.000681, p = 0.0161). The significance of the slope observed when using Bracken in the ILO cohort may be influenced by outlier sample profiles with particularly low diversity (Fig 2A).

Normalized alpha diversities generated with MetaPhlAn4 and Kraken2/Bracken were highly correlated (Pearson correlation r>= 0.78, p < 0.001 for all taxonomic levels) (Figs 2C, 2D and S3C, S3D). The results were similar when restricted to in-common taxa identified by both classifier methods (S4 Fig). Collectively, the normalized alpha diversity results suggest that both taxonomic classifiers capture consistent age associations in two cohorts at higher taxonomic levels, with inconsistent results at lower taxonomic levels.

As a sensitivity analysis, we performed an additional analysis to evaluate the normalized alpha diversity incorporating genome bins from the MetaPhlAn4 profiles (S5 Fig). The genome bins accounted for 26.7% of total relative abundances in ILO sample profiles and 17.9% of total relative abundances in the Xu sample profiles (S5A, S5B Fig). For both cohorts, the normalized alpha diversity results were consistent with our original analysis at the species level. However, we observed differences in several cases at higher taxonomic levels in the ILO cohort (S5C, S5D Fig).

In addition, an examination of taxonomic richness (total taxa identified at a given level) found that there could be disagreement in associations with age across classifiers (S6 Fig). Higher-order richness (phylum, class) increased with age in MetaPhlAn4 profiles, but was not age-associated in the Kraken2/Bracken profiles. Genus-level richness increased with age in the Xu et al. cohort for MetaPhlAn4 profiles, but was flat for Bracken profiles. Species-level richness was not age-associated in any scenario. While measures incorporating evenness (i.e., the normalized Shannon alpha diversity) tended to have similar age associations across classifiers, conclusions about richness were more sensitive to the choice of classifier.

### Age associations with beta diversity are concordant across taxonomic classifiers

**Fig 3:**
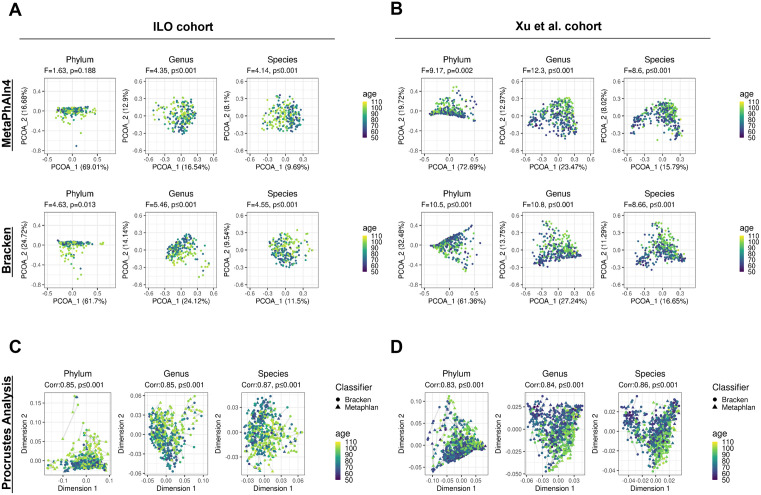
Beta diversity displays similar changes with age based on profiler approach in each cohort. **(A-B)** Principal coordinate analysis plots displaying the Bray-Curtis dissimilarities between samples, comparing across phylum, genus, and species taxonomic levels within cohorts and between cohorts and classification methods. We employed PERMANOVA to evaluate the differences in the Bray-Curtis dissimilarities between samples with the association with age, with a significance threshold set at 0.05. **(C-D)** Scores from Procrustes analysis performed on Bray-Curtis dissimilarities of samples from both classification methods within each cohort, with lines connecting the same samples. We employed Procrustian randomization (Monte Carlo) test to evaluate whether the concordance between the distances based on the taxonomic classifiers is greater than expected due to chance, with significance threshold set at 0.05.

We calculated beta diversity with Bray-Curtis dissimilarity index for each cohort and both taxonomic classifiers to examine the changes in beta diversity with age. We then performed principal coordinate analysis (PCoA) to visualize the similarities and differences between samples (Figs 3 and S7; S3, S4 Tables). The percent of variability explained by the first component of the PCoA was greater than 69% at the phylum level and decreased to less than 20% at the species level across classifiers and cohorts. The PCoA plots display greater similarity between samples of similar ages compared to samples of different ages, with statistically significant associations observed in most cases between different classifiers and cohorts (Figs 3A, 3B and S7A, S7B). One exception included differences at the phylum level in the ILO cohort, in which MetaPhlAn4 did not identify statistically significant changes in beta diversity with age (PERMANOVA F = 1.63, p = 0.188), although statistically significant differences were observed when using Bracken (PERMANOVA F = 4.63, p = 0.013) (Fig 3A).

We used Procrustes analysis to show graphically whether the dissimilarity between any pair of samples within a cohort are maintained regardless of the classifier used. To assess this further, we used a Procrustean randomization test to measure whether the correspondence across methods was greater than would be expected due to chance (Figs 3C, 3D and S7C, S7D). There was high correspondence in dissimilarities at all taxonomic levels (correlation: 0.83-0.87, p ≤ 0.001) between Bracken-based profiles versus MetaPhlAn4-based profiles in both cohorts. Additionally, Mantel tests showed high correlation among Bray-Curtis dissimilarities based on MetaPhlAn4 versus Kraken2 (correlation>0.75, p ≤ 0.001 for all taxonomic levels; Table 3). These findings were similar when limiting the analysis to in-common taxa identified by both classifier methods (S8 Fig). Overall, the beta diversity results suggest that both taxonomic classifiers capture similar community-level changes with age in both cohorts.

**Table 3:** Correlation of diversity measures between classifiers within cohort.

Cohort	Diversity Analysis^a^	Correlation Analysis^b^	Phylum^c^	Class^d^	Order^e^	Family^f^	Genus^g^	Species^h^
ILO	Alpha	Pearson	r = 0.79 p = 6.85e-48	r = 0.79 p = 2.67e-47	r = 0.79 p = 3.09e-47	r = 0.76 p = 4.14e-42	r = 0.78 p = 2.27e-45	r = 0.82 p = 6.72e-55
	Beta	Mantel	r = 0.85 p ≤ 0.001	r = 0.88 p ≤ 0.001	r = 0.88 p ≤ 0.001	r = 0.89 p ≤ 0.001	r = 0.91 p ≤ 0.001	r = 0.89 p ≤ 0.001
		Procrustes	r = 0.85 p ≤ 0.001	r = 0.86 p ≤ 0.001	r = 0.87 p ≤ 0.001	r = 0.84 p ≤ 0.001	r = 0.85 p ≤ 0.001	r = 0.87 p ≤ 0.001
Xu	Alpha	Pearson	r = 0.84 p = 5.51e-92	r = 0.86 p = 1.22e-100	r = 0.86 p = 7.14e-101	r = 0.81 p = 1.2e-79	r = 0.82 p = 1.96e-85	r = 0.80 p = 3.82e-79
	Beta	Mantel	r = 0.78 p ≤ 0.001	r = 0.79 p ≤ 0.001	r = 0.79 p ≤ 0.001	r = 0.83 p ≤ 0.001	r = 0.86 p ≤ 0.001	r = 0.86 p ≤ 0.001
		Procrustes	r = 0.83 p ≤ 0.001	r = 0.83 p ≤ 0.001	r = 0.84 p ≤ 0.001	r = 0.83 p ≤ 0.001	r = 0.84 p ≤ 0.001	r = 0.86 p ≤ 0.001

^a^ Diversity analyses performed (Alpha or Beta diversity).

^b^ Correlation analysis test performed to compare diversity measures between methods.

^c-h^ Taxonomic level at which correlation test was performed, reporting correlation (r) and p-value (p).

### Differential abundance analysis reveals benefits of a multi-classifier approach

**Fig 4:**
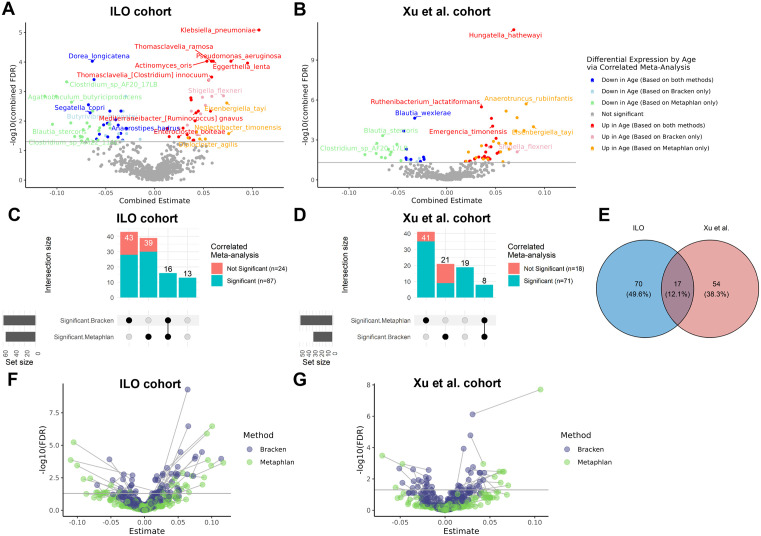
Differential abundance analysis displays shared, classifier-specific and cohort-specific differences. **(A-B)** Volcano plot of the differentially abundant species with age in each cohort. We employed linear regression models with generalized estimating equations to assess age associations with species relative abundance, and used correlated meta-analysis to generate combined p-values and combined effect estimates for species identified by both classifiers within a cohort, with significance assessed with combined FDR < 0.05. Significant differentially abundant species labelled by upregulation and downregulation with age using both taxonomic classifiers or a single classifier. **(C-D)** UpSet plots containing all species significantly associated with age via individual tests or correlated meta-analysis approach. The vertical bar heights show the number of species associated in the Bracken profiles, MetaPhlAn4 (MetaPhlAn) profiles, both, or neither based on individual tests. Blue shading shows the number of species significantly associated with age via the correlated meta-analysis approach, and red shading shows number of species significantly associated with age via individual tests that were not significant using the correlated meta-analysis approach. Of the 87 (71) species identified by the correlated meta-analysis approach in the ILO (Xu) cohorts, only 16 (8) would have been discovered by a single-profiler approach regardless of whether Bracken or MetaPhlAn4 was used. Additionally, 13 (19) age-associated species were identified in the ILO (Xu) cohorts by correlated meta-analysis that would not be identified by either Bracken or MetaPhlAn4 alone. **(E)** Venn diagram of age-associated species identified by the correlated meta-analysis approach in the ILO and Xu et al. cohorts. **(F-G)** Volcano plots of the differentially abundant species with age in each cohort based on individual tests. We employed linear regression models with generalized estimating equations to assess age associations with species relative abundance adjusting for sex and education using both classifiers within a cohort. For each taxon, the estimates and FDR values based on each classifier method are displayed with a line connecting between methods: Bracken (purple) and MetaPhlAn4 (Metaphlan) (green). For each classifier method, significance was assessed with FDR < 0.05.

To explore changes in individual taxa abundance with age based on each taxonomic classifier, we performed differential abundance analysis at the species level (Fig 4 and S5 Table). The volcano plots in Fig 4 summarize the species-level results for ILO (Fig 4A) and Xu et al. cohorts (Fig 4B). In ILO, the analysis based on MetaPhlAn4 identified 55 species whose relative abundances were associated with age, while the analysis based on Kraken2/Bracken identified 59 species associated with age at 5% FDR (Fig 4C). In Xu et al., the analysis based on MetaPhlAn4 identified 49 species whose relative abundance were associated with age, while the analysis based on Kraken2/Bracken identified 29 species associated with age at 5% FDR (Fig 4D). Use of both MetaPhlAn4 and Bracken resulted in the identification of many age-associated species that would not be found using one method alone. For instance, 43 (21) species in ILO (Xu) were age-associated in Bracken alone, which would not have been identified using MetaPhlAn4, and likewise 39 (41) species in ILO (Xu) were age-associated in MetaPhlAn4 alone (Fig 4C, 4D).

We used a correlated meta-analysis approach, AdjMaxP [27,28], to integrate results across both classifier methods while accounting for the non-independence of tests of association for taxa measured via two classifiers in the same individuals (see Methods). First, we present species-level results within each of the cohorts. Of the 43(21) species in ILO(Xu) that were significantly age-associated in the Bracken profiles but were not age-associated (or were absent) in the MetaPhlAn4 profiles, 28(9) species displayed significant association with age using the correlated meta-analysis approach (Fig 4C, 4D). Species include *Mediterraneibacter [Ruminococcus] gnavus,* linked to inflammation and aging [29,30], whose abundance in ILO was positively age-associated via Bracken and the correlated meta-analysis, and borderline but not significantly age associated (p = 0.051) via MetaPhlAn4. Of the 39(41) species in ILO(Xu) that were significantly age-associated in the MetaPhlAn4 profiles but were not age-associated (or were absent) in the Bracken profiles, 30 (35) species showed significant association with age by correlated meta-analysis (Fig 4C, 4D). This set of species include *Neglectibacter timonensis*, *Eisenbergiella tayi*, and *Clostridium_sp_AF20_17LB* that have been previously reported in association with longevity [31]. In addition, 16 (8) species in ILO (Xu) were found to be significantly associated with age by both classifier methods via individual tests, all of which were also identified by the correlated meta-analysis method (Fig 4C, 4D). Further, correlated meta-analysis also identified species that were not significantly associated with age in *either* MetaPhlAn4 or Bracken individually, but were found to be significantly age-associated in the meta-analysis (13 in ILO, 19 in Xu et al.; Fig 4C, 4D). These include species with documented age associations such as *Anaerostipes hadrus, Faecalibacterium prausnitzii,* and *M. gnavus* in the Xu et al. cohort [30,32,33].

Of the 141 total species found to be age-associated in at least one cohort by the correlated meta-analysis, we found 17 were associated with age in *both* cohorts (Fig 4E and S5 Table). Finally, we further compared species-level effects and FDR q-values between the classifier methods based on individual tests (Fig 4F, 4G). Age effect sizes tended to be larger for MetaPhlAn4 compared with Bracken, and no clear pattern was present with respect to relative FDR q-values between the classifiers.

### Benchmarking of AdjMaxP method

To evaluate the relative performance of the correlated meta-analysis approach, AdjMaxP, to identify conserved signals across inter-dependent studies (i.e., classifier profiles), we compared this method to other methods for identifying conserved features (i.e., features that are phenotype-associated in both studies), including: 1) an approach introduced by Province and Borecki [34], and 2) a “naïve” concordance approach of assigning conservation based on shared direction of effect and significance across studies. We simulated species association p-values for two studies (i.e., classifier profiles) representing varying levels of inter-study dependency, and unconserved and conserved phenotype associations of varying signal strengths, and compared the performance of the three methods (see Methods). Mean performance estimates across all metrics and conditions evaluated are reported in S6 Table for the two-study simulations based on 400 shared features. In simulations of results with no conserved and unconserved features, all methods controlled the nominal Type I error rate at 5%, with the naive method over-controlling in the case of low correlation between studies (S9 Fig, left panel). Additionally, the AdjMaxP method maintained mean specificity to conserved features >90% for all scenarios (S9 Fig). For an FDR q-value threshold of 0.05, specificity and precision were high for the AdjMaxP and naive methods, and they dropped for the Province-Borecki method as the number and signal strength of unconserved features increased, highlighting the utility of the AdjMaxP method at identifying conserved features in particular (S10A, S10B Fig). AdjMaxP’s high specificity and precision to preferentially detect conserved features came at the cost of lower sensitivity compared to Province-Borecki. However, AdjMaxP had much higher sensitivity than the naive method in most cases, except when between-study correlation was high (>0.8) (S10C Fig). For reference, the tetrachoric correlations observed in the current study were 0.376 for ILO and 0.334 for Xu et al. The area under the curve was highest for AdjMaxP across nearly all simulation scenarios considered (S10D Fig). Overall, AdjMaxP outperformed Province-Borecki and the naïve concordance approach under conditions in which unconserved features are present in the data, across various metrics.

## Discussion

### Overview

Previous benchmarking studies of taxonomic classifiers of metagenomic data have advocated for consensus methods as a way of identifying results [14,17,18]. In this report, we present an analysis of age-associated features of gut microbial communities in older and long-lived adults (50–107 years), in which we use consensus and a novel meta-analytic approach to integrate findings from two popular metagenomic taxonomic classifiers, MetaPhlAn4 and Kraken2. We performed diversity analyses in which both classifiers captured similar age-associated changes in two long-lived cohorts, with species-level variability observed across classifiers and cohorts. We also conducted a novel analysis to integrate differential abundance results between classifiers using a correlated meta-analysis approach, which identified 17 species with robust association with age across cohorts, including species that would not have been detected using one profiler alone.

### Database differences and low-abundance taxa drive profile differences between classifiers

The general observation from examining taxonomic overlap is that the choice of the taxonomic classifier affects in a substantial way the taxa identified at all levels. In our cohorts, Kraken2/Bracken identified 33–67% more species than MetaPhlAn4 (Table 1), which matches findings in previous benchmarking studies [16]. This difference did not appear to be the result of differences in numbers of species present in the databases, as Kraken2’s database had just 11% more species than MetaPhlAn4’s, and because Kraken2 identified many species that were present in the MetaPhlAn4 database but not identified by MetaPhlAn4 (Table 2). This finding agrees with previous work that characterized Kraken2/Bracken as being more sensitive and more prone to false positive calls than MetaPhlAn4 [12,15]. Eight of the top 10 abundant species overlapped between the two classifiers (S2C, S2D Fig), suggesting general agreement in quantifying the most abundant species. One possible reason for low levels of overlapping species identified is that the reference databases available for MetaPhlAn4 and Kraken2 have relatively few (~30%) species in common. These differences may be due to limitations of the approaches used by each taxonomic profiler, such as a lack of appropriate marker genes in MetaPhlAn4 or lack of species genome bins (SGBs) from metagenome-assembled genomes in the Kraken2 RefSeq databases.

### Age trends in microbiome diversity generally display correspondence across classifier methods

Despite the different microbiome compositions detected by the two tools, the downstream microbiome diversity analyses generally pointed to similar findings. For example, both classifiers identified that alpha diversity in the gut microbiome increased with age at the phylum level (Fig 2), a finding which agrees with previous work [23,35,36]. In addition, we observed strong correlations between the normalized alpha diversity scores calculated from both classifiers’ profiles. However, inconsistencies in species-level alpha diversity associations in particular highlight the existence of classifier-specific biases not only in which species are identified (Fig 2A, 2B), but in quantifying relative abundances of the detected species, with downstream implications for biological interpretations. Other studies have also found inconsistencies in age-related changes in species-level alpha diversity [24], and our observations suggest that choice of taxonomic classifier may be a factor contributing to such inconsistencies. Associations between taxonomic richness and age were found to be sensitive to the choice of classifier, which is unsurprising given the broad differences in which taxa are identified by each classifier. Investigators should be cautious when basing conclusions regarding taxonomic richness on results from a single taxonomic classifier.

Beta diversity ordination plots revealed similar relationships among samples when using MetaPhlAn4 versus Kraken2/Bracken, and comparable associations between taxonomic composition and age based on PERMANOVA (Fig 3). The significant changes in beta diversity with age observed using both classifiers have also been documented in previous studies [23,24,37,38]. Procrustes analysis and Mantel tests showed significant correspondence and high correlation of the taxonomic profiles at all levels (Fig 3C, 3D and Table 3), suggesting that despite taxon-level differences in profiles, broad phenotypic associations with taxonomic profiles may be preserved across classifiers.

### Differential abundance analyses using both classifier methods capture more age-associated taxa

Consensus and meta-analytic approaches may be especially fruitful when comparing results from phenotype associations with individual taxa (Fig 4 and S5 Table). Individual taxa are subject to false positive identifications, false negatives, and biases in quantification, and thus tools to identify robust associations are critical [16]. To synthesize evidence from across classifier methods, we applied a correlated meta-analytic procedure currently in development by our group [27,28] to combine p-values from age association tests performed on two taxonomic profiles from the same samples. In cases where only one method identified a given taxon, the p-value from the individual test was used. Of the 141 species found to be associated with age using this method, 17 were significantly age associated in both cohorts, representing the taxa with the greatest evidence for age association based on our data. The remainder of this section focuses on those 17 species (S5 Table).

Among the 17 age-associated species replicated across cohorts, many would have been missed if we used only one taxonomic classifier. Of the seven taxa that were only identified by MetaPhlAn4, *Neglectibacter timonensis*, *Eisenbergiella tayi*, and *Clostridium_sp_AF20_17LB* had previously been identified as differentially abundant in long-lived individuals compared to younger individuals across cohorts in a study that included the Xu et al. cohort and seven other cohorts [31]. For the other species identified by MetaPhlAn4 alone (*Diplocloster agilis*, *Clostridium sp_AM22_11AC*, *Blautia stercoris*, *Agathobaculum butyriciproducens)*, we did not find previously documented associations with aging or longevity in humans. However, these species have been implicated in mouse models of aging and age-related diseases, suggesting potential age-associated species for further investigation in humans. *A. butyriciproducens* and *B. stercoris* have been linked to mouse models of age-related changes in cognition and other conditions. *A. butyriciproducens* was found to decrease age-associated cognitive deficits [39] and AD-related cognitive deficits/pathology [40] in mice. *A. butyriciproducens* was found to have neuroprotective effects in mouse models of Parkinsons [41], and has been associated with decreased PET amyloid burden in humans with Alzheimer’s disease [42]. *B. stercoris* has also been used in mouse models of autism spectrum disorder to decrease behavioral deficits [43].

In addition, of the 17 hits, three were identified by Kraken2/Bracken alone. Identification of *Shigella flexneri* in Kraken2/Bracken but not in MetaPhlAn4 may be explained by the fact that *S. flexneri* and *Escherichia coli* can be hard to differentiate, with previous work showing that k-mer based methods may be more effective than marker-gene methods at differentiating the two [44,45]. The other age-associated taxa identified by Kraken2/Bracken alone were very low abundance species with no previous isolates in humans (*Butyrivibrio hungatei*, generally found in ruminant animals [46], and *Romboutsia ilealis*, isolated from the small intestine of the rat [47]). A spot-check of all forward reads that Kraken2 classified as *R. ilealis* from one sample found more 100% identity matches to *R. timonensis* (33 out of 162 reads) than to *R. ilealis* (30 out of 162 reads). A similar check of the same sample for reads assigned to *B. hungatei* found the most 100% identity matches to *Blautia luti* (133 out of 835 reads), *Blautia obium* (119 out of 835 reads), and *Blautia wexlerae* (115 out of 835 reads). It is possible Kraken2/Bracken is mis-identifying reads from human-associated close relatives as originating from these species, given the known tendency of the k-mer based tools to generate false positive calls, especially for low abundance species. While considering the union of age-associated species across profilers allows for a greater number of age associated species to be identified, it is important to consider the limitations of each classifier included, and we consider species identified by both classifiers to reflect the most likely true positive associations.

In the 17 species with robust age associations, seven were identified by both classifiers. Of these, all were significantly associated with age by the correlated meta-analysis, but not by either individual test, in at least one of the cohorts. These were generally taxa with borderline significance in the individual tests from both profiles (i.e., FDR q-values slightly greater than 0.05), where the concordance in associations across profiles resulted in a significant meta-analyzed FDR q-value. These associations include decreases with older age of species that produce anti-inflammatory short-chain fatty acids (SCFA) (e.g., *Anaerostipes hadrus*, *Faecalibacterium prausnitzii, Phocaeicola vulgatis*) and increases in pathogenic, potentially pathogenic, or pro-inflammatory species (*Enterocloster bolteae*, *Thomasclavelia [Clostridium] innocuum, Mediterraneibacter [Ruminococcus] gnavus*). Inflammation highly impacts aging, as chronic, low-grade inflammation is considered a hallmark of aging [48], and our findings support the hypothesis that shifts in microbiota with aging can contribute to increased inflammation in aging [49–51]. For example, a decrease in butyrate-producing bacteria, such as *A. hadrus and F. prautsnitzii,* has been shown to increase inflammation and is associated with inflammatory diseases such as inflammatory bowel disease [32,33]. In addition, strains of *Phocaeicola vulgatus* have also been shown to affect inflammatory diseases [52]. *M. gnavus* has been previously linked to gut dysbiosis and inflammatory conditions such as irritable bowel syndrome, relevant to aging [29,30]. In summary, the AdjMaxP meta-analytic approach we employed to combine results between the taxonomic classifiers allowed us to identify taxa with robust and biologically plausible age associations that would not have been otherwise identified.

### Limitations of the study

A limitation of this study is that, when comparing taxonomic profilers on real-world data, there is no “ground truth” or gold standard available for comparison. We focus instead on agreement and disagreement between the methods, as well as robustness of associations across cohorts and, to a degree, previous literature support for age associations. Another limitation is that our data is cross-sectional, such that we cannot make inferences about aging and longevity processes within individuals. Additionally, we restricted our analysis to taxa mappable to the NCBI database to compare and integrate across profilers, which excludes a large number of SGB-based taxonomic assignments available in MetaPhlAn4 in particular. However, the MetaPhlAn4 paper also used this approach to benchmark MetaPhlAn4 against other classifiers [2], and we examined the impact of SGBs on age-diversity associations in a sensitivity analysis. Furthermore, for brevity, we selected Bray-Curtis dissimilarity index to measure beta diversity as it is a highly utilized measure in microbiome studies of aging and longevity. Although additional factors such as diet and medication use can influence changes in the microbiome, our primary objective was to compare and integrate results across classifier methods, and a comprehensive examination of factors influencing aging and the microbiome falls outside the scope of this study. These factors will be incorporated into future work as more comprehensive metadata and samples become available in the ILO cohort. Finally, a limitation in how we treat species identified by only one classifer within the meta-analysis is that it is possible to identify taxa as significant although they may be insignificant by one method and missing by another method. However, this was observed for only 2 species in our study that became borderline significant via the correlated meta-analysis approach due to minimal differences introduced by the different FDR calculations within methods.

### Conclusions

In this study, we highlight the utility of integrating results from multiple classification methods when performing downstream analysis of microbiome data with phenotypes of interest, by analyzing data from a new Integrative Longevity Omics (ILO) cohort and a replication cohort. Previous work has highlighted the respective strengths of various taxonomic classification methods. Using two popular classifiers, we show that analyses such as Procrustes and a novel correlated meta-analytic procedure can highlight reproducible associations with biological plausibility. We also show that, regardless of the method chosen, using a single taxonomic classifier (as is standard practice in the field) results in missing potentially meaningful taxonomic associations with phenotypes.

## Methods

### Ethics statement

Centenarians, their biological offspring, and spouses of the offspring (a referent cohort) were enrolled in the ILO study between 2019 and 2024 from North America. The study was approved by the Albert Einstein College of Medicine IRB and all participants provided written informed consent.

### Experimental procedure

We provide here a brief overview of the overall approach; detailed information and methods for the recruitment of human subjects, stool sample collection, shotgun metagenomics sequencing analysis, and all statistical methods are in S1 Text.

#### Shotgun metagenomics sequencing data.

We conducted shotgun metagenomics sequencing on the gut microbiome from 220 participants of the ILO study, who were of North American/European descent and with a significant proportion of centenarians. We also obtained a publicly available shotgun metagenomics sequencing dataset from individuals of Han Chinese descent [25], in which we replicated the same processing and analysis steps performed for the ILO cohort. Detailed descriptions of these datasets are provided in S1 Text.

### Unified preprocessing and taxonomic classification

Both metagenomics datasets were processed using KneadData, and taxonomic classification was performed using both MetaPhlAn4 [2] and Kraken2 [4] via an in-house metagenomic pipeline (available at https://github.com/Integrative-Longevity-Omics/MGS_pipeline). Detailed methods describing sequence quality control, marker-gene-based classification with MetaPhlAn4, and k-mer-based classification with Kraken2 followed by Bracken are provided in S1 Text.

### Quality control pipeline after taxonomic classification

We applied identical quality control procedures to both metagenomic datasets after classification with both MetaPhlAn4 and Kraken2 to assess sample quality and exclude taxa of low abundance and potential false positives. Detailed methods are provided in S1 Text.

### Statistical analysis

#### Alpha diversity with age.

To assess changes in alpha diversity with age using different classifier approaches, we calculated a normalized alpha diversity score (see S1 Text) [53] to describe the overall heterogeneity of taxa relative abundances for each sample, and taxonomic richness as the count of taxa identified in a sample at a given taxonomic level. Linear regression model of age was used to assess differences in the normalized alpha diversity and species richness with age. Pearson correlation was used to compare sample alpha diversity between the two classification methods within each cohort. Statistical significance was determined based on p-value < 0.05.

#### Beta diversity with age.

To assess changes in beta diversity with age using different classifier approaches, we calculated the Bray-Curtis dissimilarity index between pairs of samples and performed PCoA analysis to visualize the similarities and differences between samples. PERMANOVA was used to assess differences in the Bray-Curtis dissimilarities with age. Procrustes analysis and Mantel tests were performed to evaluate the differences in the sample dissimilarities between classification methods. Statistical significance was determined based on p-value < 0.05.

#### Differential abundance analyses with age.

To assess differences in microbial relative abundances with age, we analyzed the log-transformed relative abundance values of each taxa using a generalized estimating equations model with age, sex, and education level as covariates. We calculated the FDR based on the Benjamin and Hochberg correction for multiple testing across all taxa tested. Statistical significance of taxa was determined based on FDR < 0.05.

#### Harmonization of differential abundance analyses across taxonomic classifiers.

To harmonize differential abundance analyses incorporating information across classifiers within each cohort, we used a correlated meta-analysis approach, AdjMaxP, [27,28] to generate combined p-values and estimates for all species that were identified by both MetaPhlAn4 and Kraken2. This procedure identifies signals that are conserved across non-independent sets of test results of the same features by calculating a combined p-value for each feature (i.e., species), which aggregates the nominal p-values of the differential abundance tests for each shared feature across classifiers based on the “adjusted maximum p-value” of the feature-level test. This is done by adapting a procedure for combining feature-level p-values across independent studies, in which the maximum p-value for the feature across studies is raised to the power of the number of studies. For instance, if one study had p = 0.05 for a feature, and the other had p = 0.10, the combined p-value would be 0.1^2^ (the probability of observing a p-value ≤0.10 in both studies under the null hypothesis). AdjMaxP modifies this by adjusting the power value to be an “effective number of studies,” accounting for non-independence between studies (i.e., classifiers). The adjustment is based on the tetrachoric correlation between the probit-transformed p-values. In the example above, a positive correlation between classifier measures would result in a combined p-value of 0.1 raised to a power greater than 1 but less than 2. This accounts for the non-independence of results being meta-analyzed—in this case, age associations with two measurements of species abundance performed on the same samples. For the combined effect estimates, we used the mean of the effect estimates from the two classifiers, since the sample sizes for classifiers within cohort are equivalent. For species that were identified by one classifier only, we used the p-values and effect estimates from the available classifier. To account for multiple testing across features, the false discovery rate was calculated from the combined p-values.

To assess AdjMaxP performance in identifying conserved statistical associations under inter-study dependence, we simulated association testing results based on 400 shared features and compared the results between AdjMaxP, the Province-Borecki approach, and a “naïve” concordance approach of assigning conservation based on shared direction of effect and significance across studies (see S1 Text). We evaluated the approaches based on specificity on nominal p-value thresholding of 0.05, and then specificity, sensitivity, relative precision, and area under the curve based on FDR-corrected q-value thresholding of 0.05.

Detailed methods of the correlated meta-analysis procedure and simulation-based evaluations are described in S1 Text.

## Supporting information

S1 Text Supplementary Materials and Methods. (DOCX)

S1 Table Number of taxa identified by MetaPhlAn4 and Kraken2/Bracken across taxonomic levels. (XLSX)

S2 Table Normalized alpha diversity scores for samples in each cohort based on MetaPhlAn4 and Kraken2/Bracken profiles across taxonomic levels. (XLSX)

S3 Table Beta diversity PCoA components for samples in ILO cohort based on MetaPhlAn4 and Kraken2/Bracken profiles across taxonomic levels. (XLSX)

S4 Table Beta diversity PCoA components for samples in Xu cohort based on MetaPhlAn4 and Kraken2/Bracken profiles across taxonomic levels. (XLSX)

S5 Table Differential abundance analysis results based on individual tests and correlated meta-analysis approach for each cohort. (XLSX)

S6 Table Simulation-based evalution of the correlated meta-analysis approach compared to other approaches. (XLSX)

S1 Fig Distribution of read counts and unique k-mer minimizer counts from Bracken data from ILO cohort and Xu et al. cohort. **(Top)** Scatterplots comparing the read counts (n_bracken_read) and unique k-mer minimizer counts (n_unique_minimizer) across each Bracken dataset with thresholds applied shown with red dotted lines. Three read count thresholds in log10 scale selected (0, 0.5, 1) and three unique k-mer minimizer count thresholds in log10 scale selected (0, 1, 3). **(Bottom)** Scatterplots comparing the read counts (n_bracken_read) and unique k-mer minimizer counts (n_unique_minimizer) across each Bracken dataset with lines between species representing each species’ most highly correlated species of greater n_unique_minimizer (correlations > 0.8 shown).
(TIF)

S2 Fig Comparison of taxonomic profiles across cohorts based on taxonomic classifier. **(A)** Stacked bar plot of total relative abundances across samples in the ILO cohort that make-up in-common taxa identified by both classifier methods and unique taxa specific to each method. **(B)** Stacked bar plot of total relative abundances across samples in the Xu cohort that make-up in-common taxa identified by both classifier methods and unique taxa specific to each method. **(C)** Stacked bar plot of total relative abundances across samples in the ILO cohort displaying top 10 abundant taxa present based on each classifier method. **(D)** Stacked bar plot of total relative abundances across samples in the Xu cohort displaying top 10 abundant taxa present based on each classifier method.
(TIF)

S3 Fig Age associated differences in alpha diversity across taxonomic levels using both taxonomic classifiers. **(A-B)** Scatterplots of the normalized alpha diversity score for each sample with age, comparing across class, order, and family taxonomic levels within cohorts and between cohorts and classification methods. We employed linear regression models to evaluate the association with age, with a significance threshold set at 0.05. **(C-D)** Scatterplots comparing the normalized alpha diversity scores of samples based on classification method within each cohort. We employed Pearson correlation analysis to evaluate differences in the sample normalized alpha diversity scores between methods, with significance threshold set at 0.05.
(TIF)

S4 Fig Age associated differences in alpha diversity across taxonomic levels based on in-common taxa identified by both classifiers. **(A-B)** Scatterplots of the normalized alpha diversity score for each sample with age within each cohort, comparing across taxonomic levels when using both classifiers (MetaPhlAn4 and Bracken) when restricting the analysis to the in-common taxa identified by both methods. We employed linear regression models to evaluate the association with age, with a significance threshold set at 0.05. **(C-D)** Correlation of normalized alpha diversity scores between classifier methods within each cohort. We employed Pearson correlation analysis to evaluate differences in the sample normalized alpha diversity scores between methods, with significance threshold set at 0.05.
(TIF)

S5 Fig Age associated differences in normalized alpha diversity based on MetaPhlAn4 profiles across taxonomic levels when accounting for genome bins. **(A-B)** Stacked bar plot of total relative abundances across samples in each cohort that make-up taxa with genome bins and taxa with NCBI taxonomic IDs based on MetaPhlAn4 profiles. **(C-D)** Scatterplots of the normalized alpha diversity for each sample with age, comparing across taxonomic levels within cohorts and between cohorts based on MetaPhlAn4 profiles including genome bins. We employed linear regression models to evaluate the association with age, with a significance threshold set at 0.05.
(TIF)

S6 Fig Age associated differences in richness across taxonomic levels using both taxonomic classifiers. **(A-B)** Scatterplots of the richness (total taxa at a given level) for each sample with age, comparing across taxonomic levels within cohorts and between cohorts and classification methods. We employed linear regression models to evaluate the association with age, with a significance threshold set at 0.05.
(TIF)

S7 Fig Age associated differences in beta diversity across taxonomic levels using both taxonomic classifiers. **(A-B)** Principal coordinate analysis plots displaying the Bray-Curtis dissimilarities between samples, comparing across class, order, family taxonomic levels within cohorts and between cohorts and classification methods. We employed PERMANOVA to evaluate the differences in the Bray-Curtis dissimilarities between samples with the association with age, with a significance threshold set at 0.05. **(C-D)** Scores from Procrustes analysis performed on Bray-Curtis dissimilarities of samples from both classification methods within each cohort, with lines connecting the same samples. We employed Procrustian randomization (Monte Carlo) test to evaluate whether the concordance between the distances based on the taxonomic classifiers is greater than expected due to chance, with significance threshold set at 0.05.
(TIF)

S8 Fig Age associated differences in beta diversity across taxonomic levels based on in-common taxa identified by both classifiers. **(A-B)** Principal coordinate analysis plots displaying the Bray-Curtis dissimilarities between samples in the ILO cohort and Xu cohort, comparing across taxonomic levels and classifier methods (MetaPhlAn4 and Bracken) when restricting the analysis to the in-common taxa identified by both methods. We employed PERMANOVA to evaluate the differences in the Bray-Curtis dissimilarities between samples with the association with age, with a significance threshold set at 0.05. **(C-D)** Correlation of sample dissimilarities between classifier methods within each cohort. We employed two tests: 1) Procrustian randomization (Monte Carlo) test to evaluate whether the concordance between the distances based on the taxonomic classifiers is greater than expected due to chance, with significance threshold set at 0.05. 2) Mantel test correlation analysis to evaluate the differences in the sample Bray-Curtis dissimilarities between methods, with significance threshold set at 0.05.
(TIF)

S9 Fig Comparison of specificity from simulations of two studies with no conserved features, based on an nominal p-value threshold of 0.05. Boxplots reflect the distribution of performance at p-value threshold 0.05, for 200 simulations of 400 shared features at different levels of background correlation and unconserved signal, across simulations of two studies with no conserved features. “Conserved” and “Unconserved” features refer to features for which signal was added (i.e., deviate from the null distribution) across all studies or fewer than all studies, respectively. “Proportion” and “Signal” indicate the proportion of features for which signal was added and the magnitude of added signal, respectively.
(TIF)

S10 Fig Comparison of performance metrics from simulations of two studies with 20% conserved features, based on an FDR corrected q-value threshold of 0.05. Boxplots reflect the distribution of performance at FDR threshold, 0.05, for 200 simulations of 400 shared features at different levels of background correlation and unconserved signal, across simulations of two studies with 20% conserved features. “Conserved” and “Unconserved” features refer to features for which signal was added (i.e., deviate from the null distribution) across all studies or fewer than all studies, respectively. “Proportion” and “Signal” indicate the proportion of features for which signal was added and the magnitude of added signal, respectively. **(A)** Specificity **(B)** Precision **(C)** Sensitivity **(D)** Area Under the Curve.
(TIF)

## References

[1] Knight R, Vrbanac A, Taylor BC, Aksenov A, Callewaert C, Debelius J, et al. Best practices for analysing microbiomes. Nat Rev Microbiol. 2018;16(7):410–22. doi: 10.1038/s41579-018-0029-9 29795328

[2] Blanco-Míguez A, Beghini F, Cumbo F, McIver LJ, Thompson KN, Zolfo M, et al. Extending and improving metagenomic taxonomic profiling with uncharacterized species using MetaPhlAn 4. Nat Biotechnol. 2023;41(11):1633–44. doi: 10.1038/s41587-023-01688-w 36823356 PMC10635831

[3] Milanese A, Mende DR, Paoli L, Salazar G, Ruscheweyh H-J, Cuenca M, et al. Microbial abundance, activity and population genomic profiling with mOTUs2. Nat Commun. 2019;10(1):1014. doi: 10.1038/s41467-019-08844-4 30833550 PMC6399450

[4] Wood DE, Lu J, Langmead B. Improved metagenomic analysis with Kraken 2. Genome Biol. 2019;20(1):257. doi: 10.1186/s13059-019-1891-0 31779668 PMC6883579

[5] Breitwieser FP, Baker DN, Salzberg SL. KrakenUniq: confident and fast metagenomics classification using unique k-mer counts. Genome Biol. 2018;19(1):198. doi: 10.1186/s13059-018-1568-0 30445993 PMC6238331

[6] Lu J, Breitwieser FP, Thielen P, Salzberg SL. Bracken: estimating species abundance in metagenomics data. PeerJ Comput Sci. 2017;3:e104. doi: 10.7717/peerj-cs.104 40271438 PMC12016282

[7] Lu J, Rincon N, Wood DE, Breitwieser FP, Pockrandt C, Langmead B, et al. Metagenome analysis using the Kraken software suite. Nat Protoc. 2022;17(12):2815–39. doi: 10.1038/s41596-022-00738-y 36171387 PMC9725748

[8] Kim D, Song L, Breitwieser FP, Salzberg SL. Centrifuge: rapid and sensitive classification of metagenomic sequences. Genome Res. 2016;26(12):1721–9. doi: 10.1101/gr.210641.116 27852649 PMC5131823

[9] Menzel P, Ng KL, Krogh A. Fast and sensitive taxonomic classification for metagenomics with Kaiju. Nat Commun. 2016;7:11257. doi: 10.1038/ncomms11257 27071849 PMC4833860

[10] Buchfink B, Xie C, Huson DH. Fast and sensitive protein alignment using DIAMOND. Nat Methods. 2015;12(1):59–60. doi: 10.1038/nmeth.3176 25402007

[11] Sun Z, Huang S, Zhang M, Zhu Q, Haiminen N, Carrieri AP, et al. Challenges in benchmarking metagenomic profilers. Nat Methods. 2021;18(6):618–26. doi: 10.1038/s41592-021-01141-3 33986544 PMC8184642

[12] Bradford LM, Carrillo C, Wong A. Managing false positives during detection of pathogen sequences in shotgun metagenomics datasets. BMC Bioinformatics. 2024;25(1):372. doi: 10.1186/s12859-024-05952-x 39627685 PMC11613480

[13] Breitwieser FP, Baker DN, Salzberg SL. KrakenUniq: confident and fast metagenomics classification using unique k-mer counts. Genome Biol. 2018;19(1):198. doi: 10.1186/s13059-018-1568-0 30445993 PMC6238331

[14] Jurado-Rueda F, Alonso-Guirado L, Perea-Cham-Blee TE, Elliott OT, Filip I, Rabadán R, et al. Benchmarking of microbiome detection tools on RNA-seq synthetic databases according to diverse conditions. Bioinform Adv. 2023;3(1):vbad014. doi: 10.1093/bioadv/vbad014 36874954 PMC9976984

[15] Meyer F, Fritz A, Deng Z-L, Koslicki D, Lesker TR, Gurevich A, et al. Critical Assessment of Metagenome Interpretation: the second round of challenges. Nat Methods. 2022;19(4):429–40. doi: 10.1038/s41592-022-01431-4 35396482 PMC9007738

[16] Xu R, Rajeev S, Salvador LCM. The selection of software and database for metagenomics sequence analysis impacts the outcome of microbial profiling and pathogen detection. PLoS One. 2023;18(4):e0284031. doi: 10.1371/journal.pone.0284031 37027361 PMC10081788

[17] McIntyre ABR, Ounit R, Afshinnekoo E, Prill RJ, Hénaff E, Alexander N, et al. Comprehensive benchmarking and ensemble approaches for metagenomic classifiers. Genome Biol. 2017;18(1):182. doi: 10.1186/s13059-017-1299-7 28934964 PMC5609029

[18] Ye SH, Siddle KJ, Park DJ, Sabeti PC. Benchmarking Metagenomics Tools for Taxonomic Classification. Cell. 2019;178(4):779–94. doi: 10.1016/j.cell.2019.07.010 31398336 PMC6716367

[19] Piro VC, Matschkowski M, Renard BY. MetaMeta: integrating metagenome analysis tools to improve taxonomic profiling. Microbiome. 2017;5(1):101. doi: 10.1186/s40168-017-0318-y 28807044 PMC5557516

[20] Metwally AA, Dai Y, Finn PW, Perkins DL. WEVOTE: Weighted Voting Taxonomic Identification Method of Microbial Sequences. PLoS One. 2016;11(9):e0163527. doi: 10.1371/journal.pone.0163527 27683082 PMC5040256

[21] Sundell D, Öhrman C, Svensson D, Karlsson E, Brindefalk B, Myrtennäs K, et al. FlexTaxD: flexible modification of taxonomy databases for improved sequence classification. Bioinformatics. 2021;37(21):3932–3. doi: 10.1093/bioinformatics/btab621 34469515

[22] Tian Q, Zhang P, Zhai Y, Wang Y, Zou Q. Application and Comparison of Machine Learning and Database-Based Methods in Taxonomic Classification of High-Throughput Sequencing Data. Genome Biol Evol. 2024;16(5):evae102. doi: 10.1093/gbe/evae102 38748485 PMC11135637

[23] Badal VD, Vaccariello ED, Murray ER, Yu KE, Knight R, Jeste DV, et al. The Gut Microbiome, Aging, and Longevity: A Systematic Review. Nutrients. 2020;12(12):3759. doi: 10.3390/nu12123759 33297486 PMC7762384

[24] Ghosh TS, Shanahan F, O’Toole PW. Toward an improved definition of a healthy microbiome for healthy aging. Nat Aging. 2022;2(11):1054–69. doi: 10.1038/s43587-022-00306-9 37118093 PMC10154212

[25] Xu Q, Wu C, Zhu Q, Gao R, Lu J, Valles-Colomer M, et al. Metagenomic and metabolomic remodeling in nonagenarians and centenarians and its association with genetic and socioeconomic factors. Nat Aging. 2022;2(5):438–52. doi: 10.1038/s43587-022-00193-0 37118062

[26] McIver LJ, Abu-Ali G, Franzosa EA, Schwager R, Morgan XC, Waldron L, et al. bioBakery: a meta’omic analysis environment. Bioinformatics. 2018;34(7):1235–7. doi: 10.1093/bioinformatics/btx754 29194469 PMC6030947

[27] Reed ER, Chandler KB, Lopez P, Costello CE, Andersen SL, Perls TT, et al. Cross-platform proteomics signatures of extreme old age. Geroscience. 2025;47(1):1199–220. doi: 10.1007/s11357-024-01286-x 39048883 PMC11872828

[28] Reed E, Sebastiani P. A Simple Strategy for Identifying Conserved Features across Non-independent Omics Studies. bioRxiv. 2024:2023.11.22.568276. doi: 10.1101/2023.11.22.568276 38045352 PMC10690236

[29] Jiang H, Song T, Li Z, An L, He C, Zheng K. Dissecting the association between gut microbiota and liver cancer in European and East Asian populations using Mendelian randomization analysis. Front Microbiol. 2023;14:1255650. doi: 10.3389/fmicb.2023.1255650 37789851 PMC10544983

[30] Ghosh TS, Shanahan F, O’Toole PW. The gut microbiome as a modulator of healthy ageing. Nat Rev Gastroenterol Hepatol. 2022;19(9):565–84. doi: 10.1038/s41575-022-00605-x 35468952 PMC9035980

[31] Chen S, Zhang Z, Liu S, Chen T, Lu Z, Zhao W, et al. Consistent signatures in the human gut microbiome of longevous populations. Gut Microbes. 2024;16(1):2393756. doi: 10.1080/19490976.2024.2393756 39197040 PMC11364081

[32] Zhang Q, Wu Y, Wang J, Wu G, Long W, Xue Z, et al. Accelerated dysbiosis of gut microbiota during aggravation of DSS-induced colitis by a butyrate-producing bacterium. Sci Rep. 2016;6:27572. doi: 10.1038/srep27572 27264309 PMC4893749

[33] Lenoir M, Martín R, Torres-Maravilla E, Chadi S, González-Dávila P, Sokol H, et al. Butyrate mediates anti-inflammatory effects of Faecalibacterium prausnitzii in intestinal epithelial cells through Dact3. Gut Microbes. 2020;12(1):1–16. doi: 10.1080/19490976.2020.1826748 33054518 PMC7567499

[34] Province MA, Borecki IB. A correlated meta-analysis strategy for data mining “OMIC” Scans | Biocomputing 2013. In: Biocomputing 2013. 2012. doi: 10.1142/9789814447973_0023 PMC3773990 23424128

[35] Santoro A, Ostan R, Candela M, Biagi E, Brigidi P, Capri M, et al. Gut microbiota changes in the extreme decades of human life: a focus on centenarians. Cell Mol Life Sci. 2018;75(1):129–48. doi: 10.1007/s00018-017-2674-y 29032502 PMC5752746

[36] Odamaki T, Kato K, Sugahara H, Hashikura N, Takahashi S, Xiao J-Z, et al. Age-related changes in gut microbiota composition from newborn to centenarian: a cross-sectional study. BMC Microbiol. 2016;16:90. doi: 10.1186/s12866-016-0708-5 27220822 PMC4879732

[37] Wilmanski T, Diener C, Rappaport N, Patwardhan S, Wiedrick J, Lapidus J, et al. Gut microbiome pattern reflects healthy ageing and predicts survival in humans. Nat Metab. 2021;3(2):274–86. doi: 10.1038/s42255-021-00348-0 33619379 PMC8169080

[38] Rampelli S, Soverini M, D’Amico F, Barone M, Tavella T, Monti D, et al. Shotgun Metagenomics of Gut Microbiota in Humans with up to Extreme Longevity and the Increasing Role of Xenobiotic Degradation. mSystems. 2020;5(2):e00124-20. doi: 10.1128/mSystems.00124-20 32209716 PMC7093822

[39] Go J, Maeng S-Y, Chang D-H, Park H-Y, Min K-S, Kim J-E, et al. Agathobaculum butyriciproducens improves ageing-associated cognitive impairment in mice. Life Sci. 2024;339:122413. doi: 10.1016/j.lfs.2024.122413 38219919

[40] Go J, Chang D-H, Ryu Y-K, Park H-Y, Lee I-B, Noh J-R, et al. Human gut microbiota Agathobaculum butyriciproducens improves cognitive impairment in LPS-induced and APP/PS1 mouse models of Alzheimer’s disease. Nutr Res. 2021;86:96–108. doi: 10.1016/j.nutres.2020.12.010 33551257

[41] Lee DW, Ryu Y-K, Chang D-H, Park H-Y, Go J, Maeng S-Y, et al. Agathobaculum butyriciproducens Shows Neuroprotective Effects in a 6-OHDA-Induced Mouse Model of Parkinson’s Disease. J Microbiol Biotechnol. 2022;32(9):1168–77. doi: 10.4014/jmb.2205.05032 36168204 PMC9628974

[42] Kim GH, Kim BR, Yoon H-J, Jeong JH. Alterations in Gut Microbiota and Their Correlation with Brain Beta-Amyloid Burden Measured by 18F-Florbetaben PET in Mild Cognitive Impairment Due to Alzheimer’s Disease. J Clin Med. 2024;13(7):1944. doi: 10.3390/jcm13071944 38610709 PMC11012963

[43] Sen P, Sherwin E, Sandhu K, Bastiaanssen TFS, Moloney GM, Golubeva A, et al. The live biotherapeutic Blautia stercoris MRx0006 attenuates social deficits, repetitive behaviour, and anxiety-like behaviour in a mouse model relevant to autism. Brain Behav Immun. 2022;106:115–26. doi: 10.1016/j.bbi.2022.08.007 35995237

[44] Valencia EM, Maki KA, Dootz JN, Barb JJ. Mock community taxonomic classification performance of publicly available shotgun metagenomics pipelines. Sci Data. 2024;11(1). doi: 10.1038/s41597-023-02877-7 PMC10794705 38233447

[45] Devanga Ragupathi NK, Muthuirulandi Sethuvel DP, Inbanathan FY, Veeraraghavan B. Accurate differentiation of Escherichia coli and Shigella serogroups: challenges and strategies. New Microbes New Infect. 2017;21:58–62. doi: 10.1016/j.nmni.2017.09.003 29204286 PMC5711669

[46] Kopečný J, Zorec M, Mrázek J, Kobayashi Y, Marinšek-Logar R. Butyrivibrio hungatei sp. nov. and Pseudobutyrivibrio xylanivorans sp. nov., butyrate-producing bacteria from the rumen. Int J Syst Evol Microbiol. 2003;53(Pt 1):201–9. doi: 10.1099/ijs.0.02345-0 12656174

[47] Gerritsen J, Fuentes S, Grievink W, van Niftrik L, Tindall BJ, Timmerman HM, et al. Characterization of Romboutsia ilealis gen. nov., sp. nov., isolated from the gastro-intestinal tract of a rat, and proposal for the reclassification of five closely related members of the genus Clostridium into the genera Romboutsia gen. nov., Intestinibacter gen. nov., Terrisporobacter gen. nov. and Asaccharospora gen. nov. Int J Syst Evol Microbiol. 2014;64(Pt 5):1600–16. doi: 10.1099/ijs.0.059543-0 24480908

[48] Baechle JJ, Chen N, Makhijani P, Winer S, Furman D, Winer DA. Chronic inflammation and the hallmarks of aging. Mol Metab. 2023;74:101755. doi: 10.1016/j.molmet.2023.101755 37329949 PMC10359950

[49] Biagi E, Franceschi C, Rampelli S, Severgnini M, Ostan R, Turroni S, et al. Gut Microbiota and Extreme Longevity. Curr Biol. 2016;26(11):1480–5. doi: 10.1016/j.cub.2016.04.016 27185560

[50] Cerro ED-D, Lambea M, Félix J, Salazar N, Gueimonde M, De la Fuente M, et al. Daily ingestion of Akkermansia mucciniphila for one month promotes healthy aging and increases lifespan in old female mice. Biogerontology 2021 23:1. doi: 10.1007/s10522-021-09943-w 34729669

[51] Wells C, Robertson T, Sheth P, Abraham S. How aging influences the gut-bone marrow axis and alters hematopoietic stem cell regulation. Heliyon. 2024;10(12):e32831. doi: 10.1016/j.heliyon.2024.e32831 38984298 PMC11231543

[52] Da Silva Morais E, Grimaud GM, Warda A, Stanton C, Ross P. Genome plasticity shapes the ecology and evolution of Phocaeicola dorei and Phocaeicola vulgatus. Sci Rep. 2024;14(1):10109. doi: 10.1038/s41598-024-59148-7 38698002 PMC11066082

[53] Karagiannis TT, Monti S, Sebastiani P. Cell Type Diversity Statistic: An Entropy-Based Metric to Compare Overall Cell Type Composition Across Samples. Front Genet. 2022;13:855076. doi: 10.3389/fgene.2022.855076 35464841 PMC9023789

